# Cross‐sectional study of therapy‐related expectations/concerns of patients with metastatic renal cell carcinoma and physicians in Japan

**DOI:** 10.1002/cam4.7196

**Published:** 2024-06-13

**Authors:** Go Kimura, Yasuhisa Fujii, Takahiro Osawa, Yosuke Uchitomi, Kazunori Honda, Miki Kondo, Ariko Otani, Tetsuya Wako, Daisuke Kawai, Yoshihide Mitsuda, Naotaka Sakashita, Nobuo Shinohara

**Affiliations:** ^1^ Department of Urology Nippon Medical School Hospital Tokyo Japan; ^2^ Department of Urology Tokyo Medical and Dental University Tokyo Japan; ^3^ Department of Renal and Genitourinary Surgery Hokkaido University Graduate School of Medicine Sapporo Japan; ^4^ Innovation Center for Supportive, Palliative and Psychosocial Care National Cancer Center Hospital Tokyo Japan; ^5^ Department of Clinical Oncology Aichi Cancer Center Nagoya Japan; ^6^ Department of Nursing National Cancer Center Hospital East Kashiwa Japan; ^7^ Department of Pharmacy Nippon Medical School Hospital Tokyo Japan; ^8^ Eisai Co., Ltd. Tokyo Japan; ^9^ Medilead, Inc. Tokyo Japan

**Keywords:** patient‐centricity, renal cell carcinoma, shared decision‐making, systemic therapy, treatment preference

## Abstract

**Objective:**

To achieve patient‐centricity in metastatic renal cell carcinoma (mRCC) treatment, it is essential to clarify the differences in perspectives between patients and physicians. This cross‐sectional analysis of a web survey aimed to clarify the differences in expectations and concerns between mRCC patients and physicians regarding systemic mRCC therapy in Japan.

**Methods:**

Surveys from 83 patients and 165 physicians were analyzed.

**Results:**

The top three most significant differences in expectations of systemic therapy between patients and physicians (patient‐based physician value) were “Chance of achieving treatment‐free status” (−30.1%, *p* < 0.001), “Longer survival” (+25.8%, *p* < 0.001), and “Chance of eliminating all evidence of disease” (−25.6%, *p* < 0.001). The top three most significant differences in concerns for systemic therapy between patients and physicians (patient‐based physician value) were “Lack of efficacy” (+36.1%, *p* < 0.001), “Lack of knowledge of treatment” (−28.2%, *p* < 0.001), and “Daily activities affected by side effects” (+22.3%, *p* < 0.001). Diarrhea, fatigue/malaise, and nausea/vomiting were patients' most distressing adverse events; 50.6% of patients had difficulty telling their physicians about adverse events such as fatigue, anxiety, and depression.

**Conclusions:**

This study demonstrated a gap between patients with mRCC and physicians in their expectations and concerns for systemic therapy. Japanese patients with mRCC suffer from a number of adverse events, some of which are not shared with physicians. This study highlights the importance of communicating well with patients in clinical practice to achieve patient‐centricity in systemic treatment for mRCC.

## Graphical abstract

Patient‐centric treatment of metastatic renal cell carcinoma (mRCC) requires understanding the treatment expectations and concerns of patients and their physicians. In this cross‐sectional study, we analyzed web surveys from 83 mRCC patients and 165 treating physicians and found significant discrepancies in their treatment perspectives. This study highlights that improvements in communication between mRCC patients and their physicians are needed to achieve appropriate patient‐centric care.

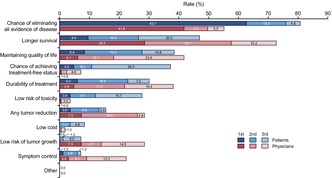

## 1 INTRODUCTION

In developed countries, renal cell carcinoma (RCC) incidence has doubled over the past decade.^1^, ^2^ In Japan, over 21,000 patients were diagnosed with RCC in 2019.^3^ The first‐line systemic treatment for metastatic RCC (mRCC) is rapidly evolving. With the introduction of immuno‐oncology (IO), IO‐based combination therapies such as IO/IO and IO/tyrosine kinase inhibitors (TKIs) have dramatically improved progression‐free survival, overall survival, and objective response rates for mRCC compared with sunitinib monotherapy.^4^, ^5^, ^6^, ^7^, ^8^, ^9^, ^10^, ^11^ However, the available IO‐combination therapies and TKI monotherapies have different efficacy and safety profiles and impact on quality of life (QOL). Japan has a vast selection of drugs covered under its universal health insurance system; as a result, there is an increasing awareness of the importance of patient‐centricity in cancer treatments.

To achieve patient‐centricity in mRCC treatment, it is essential to clarify the differences in perspectives between patients and physicians regarding efficacy, safety, and QOL.^12^ Furthermore, it is important to provide patients with appropriate information about cancer treatment options and their potential side effects. However, the levels of cancer literacy vary from country to country and region to region, and some populations lack systematic information about cancer treatment.^13^ It has also been suggested that inequalities in the shared decision‐making (SDM) process among patients of different racial and ethnic backgrounds affect the quality of healthcare they receive.^14^ Several barriers to SDM in oncology have been identified, including uncertainty in the treatment decision, concerns regarding adverse effects, and poor physician communication.^15^ Because of the wide range of treatment options available for mRCC, it is crucial to provide individualized treatment plans for each patient. Implementing SDM can aid patients in comprehending the purpose of their treatment choices.

It is also important to properly communicate the benefits and risks of treatment and identify treatment preferences by understanding the patient's treatment expectations.^16^ Both patient and physician perspectives are important and should be openly discussed, and may include factors such as patients' expectation of treatment outcomes and treatment‐related concerns.^16^ In a survey of physicians and mRCC patients, respondents indicated that the probability of living ≥3 years was the most important attribute of mRCC treatment.^17^ Similarly, a study of advanced RCC patients and physicians reported that efficacy (survival gain) was the most important determinant of treatment preference, followed by the health‐related quality of life (HRQOL) provided by the treatment.^18^

During the treatment of mRCC, there are differences between patient‐reported adverse reactions and those reported in clinical trials.^19^ This suggests that healthcare providers may not always be fully aware of the treatment preferences or concerns of mRCC patients. Specifically, no studies have distinguished between expectations and concerns as attributes of treatment preferences. Although it has been suggested that the SDM process varies by country and ethnic background,^13^, ^14^ no reports in Japan have examined the preferences of mRCC patients for appropriate SDM. In particular, the adverse event profiles of mRCC patients, considered the most distressing and challenging to communicate to physicians in clinical practice, have not been investigated. Therefore, this survey aimed to clarify the expectations and concerns of mRCC patients and physicians regarding systemic therapy in Japan. Furthermore, we intended to clarify the adverse events causing the most distress and those challenging to communicate to the physicians for mRCC patients.

## 2 MATERIALS AND METHODS

### 2.1 Study design and participants

This was a cross‐sectional web survey study conducted in Japan. The target population included patients with mRCC who received systemic cancer therapy and physicians who treated RCC patients.

Patients who agreed to participate in the web questionnaire survey and were eligible for enrollment in this study were aged ≥20 years, with advanced renal cell cancer, living in Japan, and had received or were undergoing molecular targeted therapy or immune checkpoint inhibitor therapy (including combination therapy) for mRCC. Eligible physicians were those who agreed to participate in the web questionnaire survey, were treating RCC patients, and had prescribed systemic therapy for RCC.

Patients and their families who had been associated with healthcare professionals, pharmaceutical companies or marketing companies, patients with multiple cancers, and physicians who had not prescribed systemic therapy for RCC were excluded from the study.

### 2.2 Survey implementation

All data collection by web‐based survey was conducted between May 2022 and June 2022. The following survey panels and an online peer support group were used: Research Panel, Inc., Rakuten Insight, Inc., 3H Holdings Inc., Nikkei Business Publications, Inc., and General Incorporated Association Cancer Parents.

Questionnaires for mRCC patients and physicians were developed for this study based on questionnaires used in similar studies published previously.^20^, ^21^, ^22^ Attributes of expectations and concerns were investigated separately.

The questionnaires for mRCC patients and physicians comprised the following sections.

#### 2.2.1 Participant characteristics

For mRCC patients, demographic information collected included age, gender, treatment history, and duration of administration.

For physicians, demographic information collected included years of clinical experience as a physician, years of clinical experience in treating patients with RCC, gender, clinical department, and the predominant type of hospital they were working in.

#### 2.2.2 Expectations and concerns of systemic therapy

We investigated mRCC patients' expectations of systemic therapy according to 11 attributes related to effectiveness, toxicity, QOL, and cost. Patients completed the questionnaire, ranking the three most important attributes from first to third place. To investigate what mRCC patients expect from systemic therapy from a physician's perspective, the same attributes as mRCC patients were used, and the physicians ranked the three most important, from first to third place.

For mRCC patients, we investigated patients' concerns about systemic therapy based on nine attributes related to effectiveness, toxicity, financial burden, knowledge and information about systemic therapy, and communication. Patients ranked each attribute from first to third place. From the physician's perspective, we investigated the concerns of mRCC patients regarding systemic therapy based on the same nine attributes used for evaluating mRCC patients. Physicians ranked each attribute from first to third place.

#### 2.2.3 Adverse events (only mRCC patients)

To investigate the most distressing adverse events of systemic therapy, 28 adverse event‐related symptoms associated with the systemic therapy currently used, including fever, anorexia, fatigue, pain, constipation, diarrhea, skin rashes, hair loss, insomnia, anxiety, and vomiting, were evaluated. Patients filled out the questionnaire and ranked the top three symptoms from first to third. Then, the top 10 most common adverse event‐related symptoms out of the top three ranked by each patient were extracted, and the percentage of these ranked first through third was evaluated.

To investigate adverse events that were difficult to communicate to the physician during systemic therapy, the same 28 symptom items using an examination of distressing adverse events were used. Patients filled out the questionnaire ranking the top three symptoms from first to third place. The top three adverse events listed by each patient were then tabulated, and the top 10 most common were evaluated.

In addition, we investigated why the patients answered that they had an adverse event that was difficult to communicate to their physician based on 14 attributes. The patients selected all applicable answers among the 14 attributes.

### 2.3 Endpoints

The primary endpoint was the differences in expectations of systemic therapy between mRCC patients and physicians. The key secondary endpoints were the differences in concerns about systemic therapy between mRCC patients and physicians, the most distressing adverse events for mRCC patients, and adverse events that mRCC patients found difficult to communicate to physicians.

### 2.4 Sample size and data analysis

The sample size of each target population was set based on the number of patients and physicians registered in the web‐based survey panels. The target numbers were 95 and 150 for patients and physicians, respectively. Fisher's exact test was used for assessing the independence between patients and physicians. R (version 4.2.0, R Foundation for Statistical Computing, Vienna, Austria) was used to perform all the statistical analyses. Statistical significance was set at a nominal *p* < 0.05.

### 2.5 Ethical considerations

All methods were carried out per the principles and guidelines of the Declaration of Helsinki, and the study protocol was approved by the Research Ethics Committee of The Japanese Association for the Promotion of State‐of‐the‐Art in Medicine. All data collected were kept confidential and only used for research purposes. All participants provided written informed consent.

## 3 RESULTS

### 3.1 Participant characteristics

Between May and June 2022, 101 patients with mRCC were screened for this study. Six patients had contradictory answers, and 12 met the study exclusion criteria; thus, data from 83 patients were analyzed (Table 1; Figure S1). Additionally, 183 physicians were screened for this study. Because of contradictory answers, 18 physicians were excluded, and the remaining 165 physicians were included in the analysis. In this study, there was no direct medical relationship between the patient and the physician.

**TABLE 1:** Demographic and clinical characteristics of the participants.

Characteristic	Value
Patients, *n*	83
Age (mean ± SD), years	44.2 ± 12.2
Gender, *n* (%)	
Man	53 (63.9)
Woman	30 (36.1)
Treatment history, *n* (%)	
Systemic therapy	83 (100)
Ongoing	77 (92.8)
Discontinued or interrupted	6 (7.2)
Surgery	31 (37.3)
Radiation therapy	3 (3.6)
Duration of systemic therapy, *n* (%)	
Less than 6 months	25 (30.1)
6 months to 2 years	29 (34.9)
More than 2 years	29 (34.9)
Physicians, *n*	165
Career as physician (mean ± SD), years	18.1 ± 7.6
Experience in RCC treatment (mean ± SD), years	15.8 ± 7.4
Gender, *n* (%)	
Man	152 (92.1)
Woman	12 (7.3)
Other	1 (0.6)
Specialty, *n* (%)	
Urology	103 (62.4)
Medical oncology	50 (30.3)
Renal transplant surgery	12 (7.3)
Main type of hospital they were working in, *n* (%)	
University hospital	35 (21.2)
Public hospital	53 (32.1)
General hospital	72 (43.6)
Clinic	5 (3.0)

Abbreviations: RCC, renal cell carcinoma; SD, standard deviation.

mRCC patients had a mean (standard deviation) age of 44.2 ± 12.2 years, and 63.9% were male. All patients received systemic treatment for mRCC, and in 92.8% of patients, systemic treatment was ongoing. The duration of systemic therapy was less than 6 months in 30.1% of patients, 6 months to 2 years in 34.9%, and more than 2 years in 34.9%. The most frequent physician specialty was urology (62.4%); other common specialties were medical oncology (30.3%) and renal transplant surgery (7.3%). Most physicians were male (92.1%).

### 3.2 Expectations of systemic therapy

**FIGURE 1:**
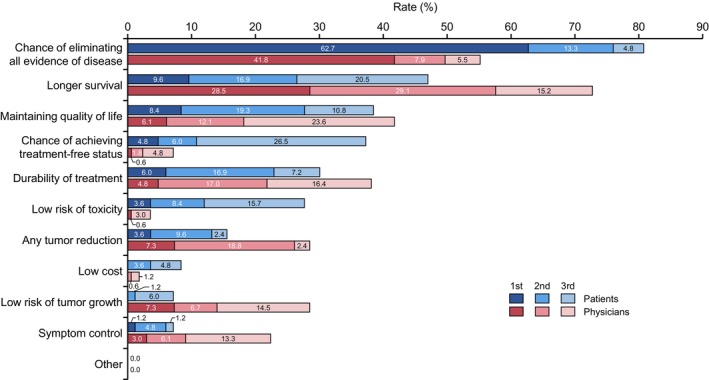
Differences between mRCC patients and physicians in expectations of systemic therapy. Horizontal bars show totals from first to third place for each mRCC patient and physician. In the figure, each item is in order of the most frequent responses selected by the patients.

Regarding expectations of systemic treatment, patients were asked, “What are your top three expectations (goals of treatment) for drug treatment of mRCC?” The physicians were also asked, “What do you think are the top three expectations (goals of treatment) of the patient for drug treatment of mRCC?” Among the 11 attributes of expectations for systemic therapy, the most important attribute for mRCC patients was “Chance of eliminating all evidence of disease” (80.8%), followed by “Longer survival” (47.0%), “Maintaining QOL” (38.5%), “Chance of achieving treatment‐free status” (37.3%), and “Durability of treatment” (30.1%) (Figure 1). The percentages of each first expectation for systemic therapy by patients are also shown (Figure S2A).

For physicians, the most important attribute of patient's expectations regarding systemic therapy was “Longer survival” (72.8%), followed by “Chance of eliminating all evidence of disease” (55.2%), “Maintaining QOL” (41.8%), “Durability of treatment” (38.2%), “Any tumor reduction” (28.5%), and “Low risk of tumor growth” (28.5%). The percentages of each first expectation for systemic therapy by physicians are also shown (Figure S2B).

The top five most significant differences between patients and physicians were (patient‐based physician value) “Chance of achieving treatment‐free status” (−30.1%, *p* < 0.001), “Longer survival” (+25.8%, *p* < 0.001), “Chance of eliminating all evidence of disease” (−25.6%, *p* < 0.001), “Low risk of toxicity” (−24.1%, *p* < 0.001), and “Low risk of tumor growth” (+21.3%, *p* < 0.001) (Table S1). Other differences between them were as follows: “Symptom control” (+15.2%, *p* = 0.0024), “Any tumor reduction” (+12.9%, *p* = 0.028), “Durability of treatment” (+8.1%, *p* = 0.26), Low cost (−6.6%, *p* = 0.018), “Maintaining quality of life” (+3.3%, *p* = 0.68), and “Other” (0.0%, *p* = 1.0).

### 3.3 Concerns about systemic therapy

**FIGURE 2:**
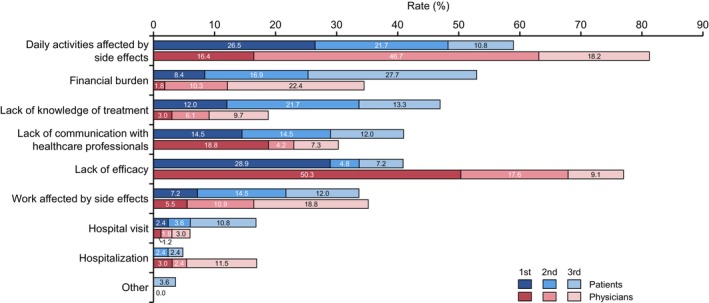
Differences between mRCC patients and physicians in concerns about systemic therapy. Horizontal bars show totals from first to third place for each mRCC patient and physician. In the figure, each item is in order of the most frequent responses selected by the patients.

Regarding concerns about systemic treatment, patients were asked, “What are the top three concerns you have about drug treatment for mRCC?” The physicians were also asked, “What do you think are the top three concerns that patients have about drug treatment of mRCC?” The most important concern for systemic therapy of mRCC for patients was “Daily activities affected by side effects” (59.0%), followed by “Financial burden” (53.0%), “Lack of knowledge of treatment” (47.0%), “Lack of communication with healthcare professionals” (41.0%), and “Lack of efficacy” (40.9%) (Figure 2). In addition, the percentages of each first concern for systemic therapy by patients are also shown (Figure S3A).

For physicians, the most important patient concern was “Daily activities affected by side effects” (81.3%), followed by “Lack of efficacy” (77.0%), “Work affected by side effects” (35.2%), “Financial burden” (34.5%), and “Lack of communication with healthcare professionals” (30.3%). The percentages of each first concern for systemic therapy by patients are also shown (Figure S3B).

The top five most significant differences between patients and physicians (patient‐based physician value) were “Lack of efficacy” (+36.1%, *p* < 0.001), “Lack of knowledge of treatment” (−28.2%, *p* < 0.001), “Daily activities affected by side effects” (+22.3%, *p* < 0.001), “Financial burden” (−18.5%, *p* = 0.0062), and “Hospitalization” (+12.1%, *p* = 0.0081) (Table S2). Other differences between them (patient‐based physician value) were as follows: “Hospital visit” (−10.8%, *p* = 0.011), “Lack of communication with healthcare professionals” (−10.7%, *p* = 0.12), “Other” (−3.6%, *p* = 0.037), and “Work affected by side effects” (+1.5%, *p* = 0.89).

### 3.4 Distressing adverse events for patients

**FIGURE 3:**
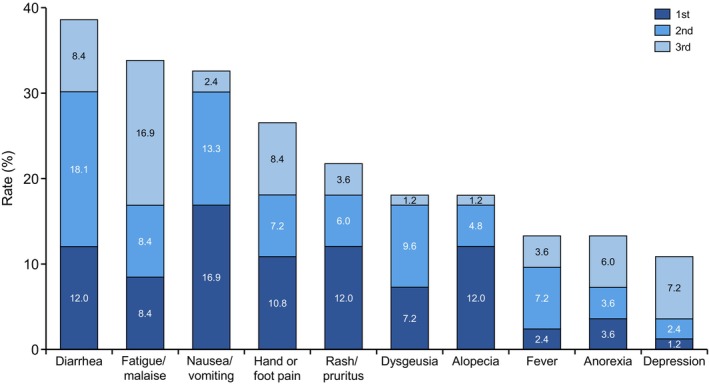
Most distressing adverse events for mRCC patients. Vertical bars show totals from first to third place for mRCC. In the figure, each item is in order of most frequent responses selected by the patients.

Regarding adverse events, patients were asked, “What are the top three most distressing adverse events or symptoms of drug treatment for mRCC?” The 10 most distressing adverse events for mRCC patients were diarrhea (38.5%), fatigue/malaise (33.7%), nausea/vomiting (32.6%), hand or foot pain (26.4%), rash/pruritus (21.6%), dysgeusia (18.0%), alopecia (18.0%), fever (13.2%), anorexia (13.2%), and depression (10.8%) (Figure 3). The percentages of each first adverse event for systemic therapy by patients are also shown (Figure S4).

**FIGURE 4:**
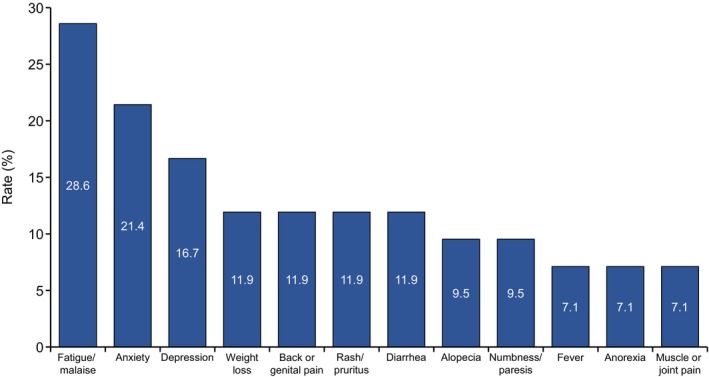
Adverse events that are difficult for mRCC patients to communicate to physicians. Of the patients who experienced adverse events, 50.6% of the patients experienced adverse events that were difficult to communicate to their physicians.

Additionally, patients were asked, “Are there any adverse events or symptoms of drug treatment for mRCC that you have difficulty communicating to your physician?” For 50.6% of the patients, some adverse events were difficult to communicate to the physicians, including fatigue/malaise (28.6%), anxiety (21.4%), depression (16.7%), weight loss (11.9%), back or genital pain (11.9%), rash/pruritus (11.9%), diarrhea (11.9%), alopecia (9.5%), numbness/paresis (9.5%), fever (7.1%), anorexia (7.1%), and muscle or joint pain (7.1%) (Figure 4).

**FIGURE 5:**
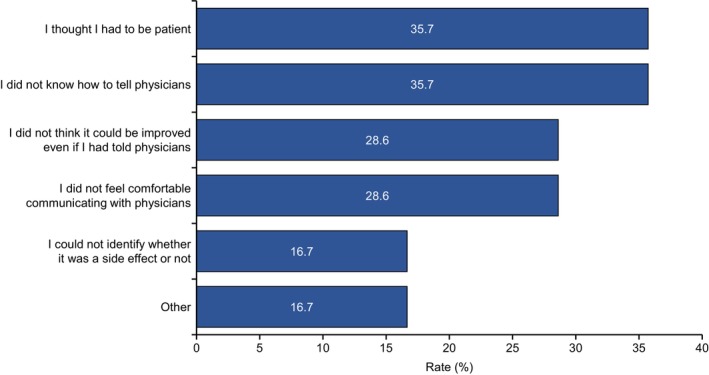
Reasons why mRCC patients have difficulty in communicating adverse events to their physicians.

Patients were further asked, “Why was it difficult to tell your physician about the adverse events or symptoms?” Reasons why it was difficult for patients to tell their physicians about adverse events included the following: “I thought I had to be patient” (35.7%), “I did not know how to tell physicians” (35.7%), “I did not think it could be improved even if I had told physicians” (28.6%), “I did not feel comfortable communicating with physicians” (28.6%), “I could not identify whether the symptom was a side effect or not” (16.7%), and “Other” (16.7%) (Figure 5).

## 4 DISCUSSION

This study demonstrated a gap between patients with mRCC and physicians in their expectations and concerns for systemic therapy. Japanese patients with mRCC suffer from a range of adverse events, some of which are not revealed to their physicians. To the best of our knowledge, this is the first study to investigate the differences in treatment perspectives between mRCC patients and physicians treating mRCC patients in Japan, intending to optimize SDM in mRCC. We also elucidated details in a setting with a wide range of mRCC treatment options, especially by distinguishing between expectations and concerns. The study also identified undesirable adverse events for mRCC patients and those difficult to communicate with healthcare providers.

The survey results revealed the attributes that mRCC patients and physicians expect from pharmacotherapy. The most important attribute for patients was the “Chance of eliminating all evidence of disease”, while for physicians, the most important was “Longer survival.” In previous reports, González et al. reported that living ≥3 years was the most important attribute for both mRCC patients and physicians,^17^ and Fernández et al. reported that the most important attribute was survival gain for both mRCC patients and physicians.^18^ In the KCCure research, the chance of eliminating all evidence of disease (complete response) as a treatment goal was recognized by more than half of the RCC patients as the most important desire or outcome they wanted to see from treatment.^21^ Other studies of preferences for mRCC patients have also reported that prolonged progression‐free survival is the most important attribute.^23^, ^24^, ^25^

In this study, “Chance of eliminating all evidence of disease” and “Longer survival” were investigated as different attributes, and the results indicated that patients placed more importance on the “Chance of eliminating all evidence of disease.” This is likely because the patients considered the “Chance of eliminating all evidence of disease” a cure and an early and clear indicator of treatment efficacy, which they hoped would lead to prolonged survival. Conversely, physicians considered “Longer survival” the most important aspect of treatment and tumor shrinking as part of the treatment process. Physicians understand that expecting a cure for mRCC may be unrealistic. Therefore, both patients and physicians may expect “Longer survival” from mRCC treatment, and this result is consistent with previous studies.^17^, ^18^, ^20^ Based on SDM, patients and physicians need to communicate regarding how the patients want to be treated, and treatment should be personalized for each patient's needs. The SHARE Approach was developed as a generalized SDM approach.^26^ In this approach, steps of letting patients know that there are options available, guiding the patient through the benefits and risks of each option, and assessing the patient's values and preferences are important for mRCC, which has diverse treatment options.

Most patients in this study were undergoing treatment during the survey, and their experience with adverse events is likely reflected in their answers. To the best of our knowledge, this survey revealed for the first time that both mRCC patients and their physicians consider “Daily activities affected by side effects” to be the most important concern in pharmacotherapy. A recent survey of mRCC patients reported that approximately 89% of participants suffered from adverse events. Although most patients reported adverse events to their physicians, roughly half did not encounter any improvement in symptoms.^19^ Indeed, in the present study, some patients did not feel that communication with their physicians about adverse events could lead to symptom improvement.

The concern of “Lack of knowledge of treatment” was remarkably different between physicians and mRCC patients in this study. A previous study of RCC patients reported that patients who received appropriate information about drug treatment tended to choose more effective treatments, even in the presence of potentially serious toxicities.^23^ These findings demonstrate that providing patients with accurate information about mRCC treatment and its potential side effects, which improves treatment literacy, is crucial for achieving patient‐centricity.^13^

The present survey findings suggest that mRCC patients are still concerned about whether they have sufficient information about their treatment and understand it. In planning for SDM, it is necessary to consider these background factors. To provide better healthcare, healthcare providers need to have a good understanding of treatment and potential side effects. Additionally, they should be able to communicate this information effectively to their patients based on the patient's expectations and concerns.

Adverse events affect the patient's HRQOL, especially in recent mRCC treatments, where the combination of IO + IO or IO + TKI is the first choice, and both immune‐related and TKI‐induced adverse events must be rapidly detected and then appropriately managed.^27^ A previous study of mRCC patients showed that fatigue, pain, nausea/vomiting, and dyspnea are significantly associated with lower HRQOL;^28^ the adverse events that patients found distressing that were identified in this study are generally consistent with the results of that previous study. This study showed that fatigue/malaise is an adverse event that patients want to avoid and is difficult for them to communicate. Fatigue is a problematic symptom to report to physicians,^29^ suggesting that its impact on patients may be underestimated.

Emotional symptoms such as anxiety and depression were also listed as adverse events that were difficult to communicate to healthcare providers. In a previous study of RCC patients, including mRCC, 27% needed psychosocial care, with the main issues being anxiety (32%), pain (27%), nervousness (26%), and sadness, worry, and sleeping difficulties (20%, respectively).^30^ An International Kidney Cancer Coalition survey reported that 96% of patients said psychosocial impact was the most important factor. However, only 50% disclosed this to their healthcare team.^31^ Healthcare professionals may not always capture such emotional symptoms, and this study supports these trends.

To appropriately screen for adverse events in patients with mRCC, multidisciplinary management that includes nurses, pharmacists, and physicians can be effective.^32^ In a survey of healthcare providers, including physicians and nurses involved in the treatment of mRCC, healthcare providers with 3–10 years of practice (37%) reported inadequate skills in assessing patients' tolerance of adverse effects. Promoting multidisciplinary care in managing immune‐related adverse events is a challenge, and breakdowns were observed in 46% of healthcare providers, particularly in monitoring adverse events and treatment adherence.^33^ Therefore, to achieve patient‐centric therapy for mRCC, it is important to improve skills and patient collaboration with healthcare providers in monitoring the adverse events of mRCC treatment. In addition to multidisciplinary collaboration, adverse event assessment tools can be used to monitor adverse events that are difficult for patients to communicate. For example, a questionnaire called the Cancer Fatigue Scale has been developed for monitoring fatigue,^34^ which was the most difficult adverse event for patients to report in this study. This is a tool that allows patients to quantify the presence and degree of fatigue by answering 15 questions. Because difficulty in explaining the degree of adverse events was one of the most common reasons for difficulty in communicating adverse events in this study, these tools could be used in daily practice for better monitoring and communication.

This study has some limitations. The mean age of the patients was 44.2 years, which was lower than the mean age at diagnosis in the United States (64.5 years)^2^ and worldwide (approximately 75 years),^1^ and much younger than the mean age of mRCC patients in Japan (66 years).^35^ It is possible that the age differences observed in this study can be attributed to the fact that the research was conducted online.^36^ Many of the patients enrolled still work, raise children, and have active lifestyles, so they may represent a patient demographic with high expectations for maintaining daily routines and a better long‐term prognosis. Additionally, the number of mRCC patients was limited, so further studies on this subject should be conducted with larger patient samples. The questionnaire used in this study is not validated. In addition, we did not investigate treatment line, and how it affects patients' treatment expectations or concerns. The study was cross‐sectional and did not include a follow‐up period. Furthermore, the timing of responses varied among respondents for both patients and physicians.

In conclusion, this study highlights the importance of communicating well with patients in clinical practice to achieve patient‐centricity in systemic treatment for mRCC. Furthermore, increasing treatment knowledge and skills in monitoring adverse events is necessary to achieve this goal.

## AUTHOR CONTRIBUTIONS

**Go Kimura:** Conceptualization (lead); writing – original draft (equal); writing – review and editing (equal). **Yasuhisa Fujii:** Conceptualization (lead); writing – original draft (equal); writing – review and editing (equal). **Takahiro Osawa:** Conceptualization (equal); writing – review and editing (equal). **Yosuke Uchitomi:** Conceptualization (equal); writing – review and editing (equal). **Kazunori Honda:** Conceptualization (equal); writing – review and editing (equal). **Miki Kondo:** Writing – review and editing (equal). **Ariko Otani:** Writing – review and editing (equal). **Tetsuya Wako:** Writing – review and editing (equal). **Daisuke Kawai:** Conceptualization (lead); investigation (equal); methodology (equal); writing – original draft (equal); writing – review and editing (equal). **Yoshihide Mitsuda:** Conceptualization (equal); investigation (equal); methodology (lead); writing – review and editing (equal). **Naotaka Sakashita:** Data curation (equal); formal analysis (equal); writing – review and editing (equal). **Nobuo Shinohara:** Conceptualization (equal); supervision (equal); writing – review and editing (equal).

## FUNDING INFORMATION

Participant recruitment, surveillance, and analysis of participant data for this study were funded by Eisai Co., Ltd. Medical writing was performed by Infront Medical Publications, and editorial assistance was provided by Keyra Martinez Dunn, MD, of Edanz (www.edanz.com), with funding from Eisai Co., Ltd.

## CONFLICT OF INTEREST STATEMENT

GK has received lecture fees from Bristol‐Myers Squibb K.K. YF is an editorial board member of Cancer Science; has received lecture fees from Takeda Pharmaceutical Co., Ltd., Ono Pharmaceutical Co., Ltd., Merck Biopharma Co., Ltd., MSD K.K., AstraZeneca K.K., Astellas Pharma Inc., Janssen Pharmaceutical K.K., and Eisai Co., Ltd.; and has received research grants from Takeda Pharmaceutical Co., Ltd. TO has received lecture fees from Ono Pharmaceutical Co., Ltd. and Takeda Pharmaceutical Co., Ltd. KH has received research grants from Pfizer Japan Inc. TW has received lecture fees from Chugai Pharmaceutical Co., Ltd., Taiho Pharmaceutical Co., Ltd., Ono Pharmaceutical Co., Ltd., Daiichi Sankyo Co., Ltd., Nippon Kayaku Co., Ltd., Kyowa Kirin Co., Ltd., Ono Pharmaceutical Co., Ltd., and Eisai Co., Ltd. N Shinohara has received lecture fees from Takeda Pharmaceutical Co., Ltd., Ono Pharmaceutical Co., Ltd., Bristol‐Myers Squibb K.K., Merck Biopharma Co., Ltd., Pfizer Japan Inc, MSD K.K., and Eisai Co., Ltd.; has received research funding from MSD K.K., Bristol‐Myers Squibb K.K., Pfizer Japan Inc, and Ono Pharmaceutical Co., Ltd.; and has received research grants from Ono Pharmaceutical Co., Ltd. and Pfizer Japan Inc. DK and YM are employees of Eisai Co., Ltd., and N Sakashita is an employee of Medilead Inc. YU, MK, and AO have no conflicts of interest to disclose.

## ETHICS STATEMENT

*Approval of the Research Protocol by an Institutional Reviewer Board*: The study was compliant with the Declaration of Helsinki and local requirements for registries. *Informed consent*: Written informed consent was obtained from patients or family members in case of communication disorders (i.e., aphasia) or cognitive impairment. *Registry and the registration number of the study/trial*: The trial was registered in the Japan Registry of Clinical Trials under the identifier jRCT1040220016. *Animal studies*: N/A.

## Supporting information


Data S1.

## Data Availability

The datasets generated during and/or analyzed during the current study are not publicly available but are available from the corresponding author on reasonable request.
