# Supplementary material for: Cross‐sectional study of therapy‐related expectations/concerns of patients with metastatic renal cell carcinoma and physicians in Japan
Source: Cancer Med. 2024 Jun 13;13(11):e7196. doi: 10.1002/cam4.7196 (PMC11176571; doi:10.1002/cam4.7196)
Supplement: Supplementary file 1 — Data S1. [file CAM4-13-e7196-s001.docx]

Supplementary Table S1: Differences between patients and physicians regarding expectations for systemic therapy

| Attribute | *P* value |
| --- | --- |
| Chance of eliminating all evidence of disease | <0.001 |
| Longer survival | <0.001 |
| Maintaining quality of life | 0.68 |
| Chance of achieving treatment-free status | <0.001 |
| Durability of treatment | 0.26 |
| Low risk of toxicity | <0.001 |
| Any tumor reduction | 0.028 |
| Low cost | 0.018 |
| Low risk of tumor growth | <0.001 |
| Symptom control | 0.0024 |
| Other | 1.0 |

**Supplementary Table S2: Differences between patients and physicians regarding concerns about systemic therapy**

| Attribute | *P* value |
| --- | --- |
| Daily activities affected by side effects | <0.001 |
| Financial burden | 0.0062 |
| Lack of knowledge of treatment | <0.001 |
| Lack of communication with healthcare professionals | 0.12 |
| Lack of efficacy | <0.001 |
| Work affected by side effects | 0.89 |
| Hospital visit | 0.011 |
| Hospitalization | 0.0081 |
| Other | 0.037 |

**Supplementary Figure S1: Flowchart showing recruitment of patients and physicians**


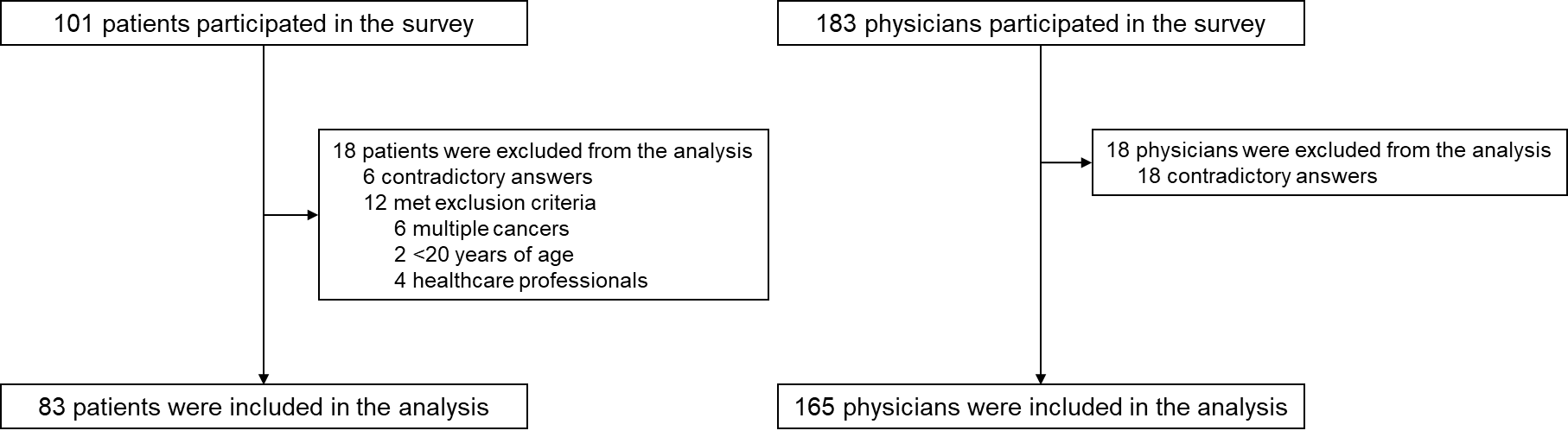


Supplementary Figure S2: Expectations of systemic therapy for patients (A) and physicians (B) (only first-ranked responses)

**
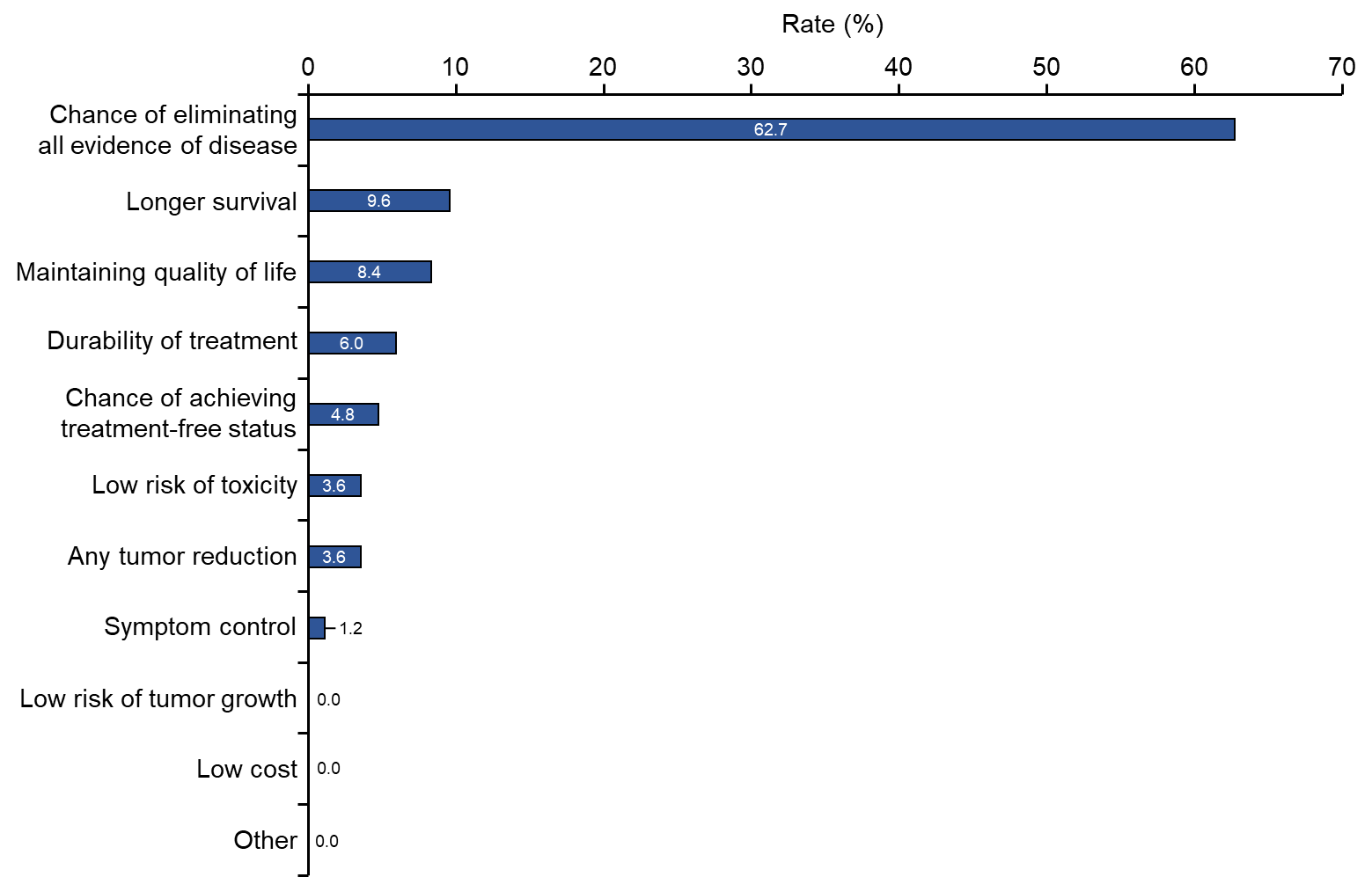
A.**

**
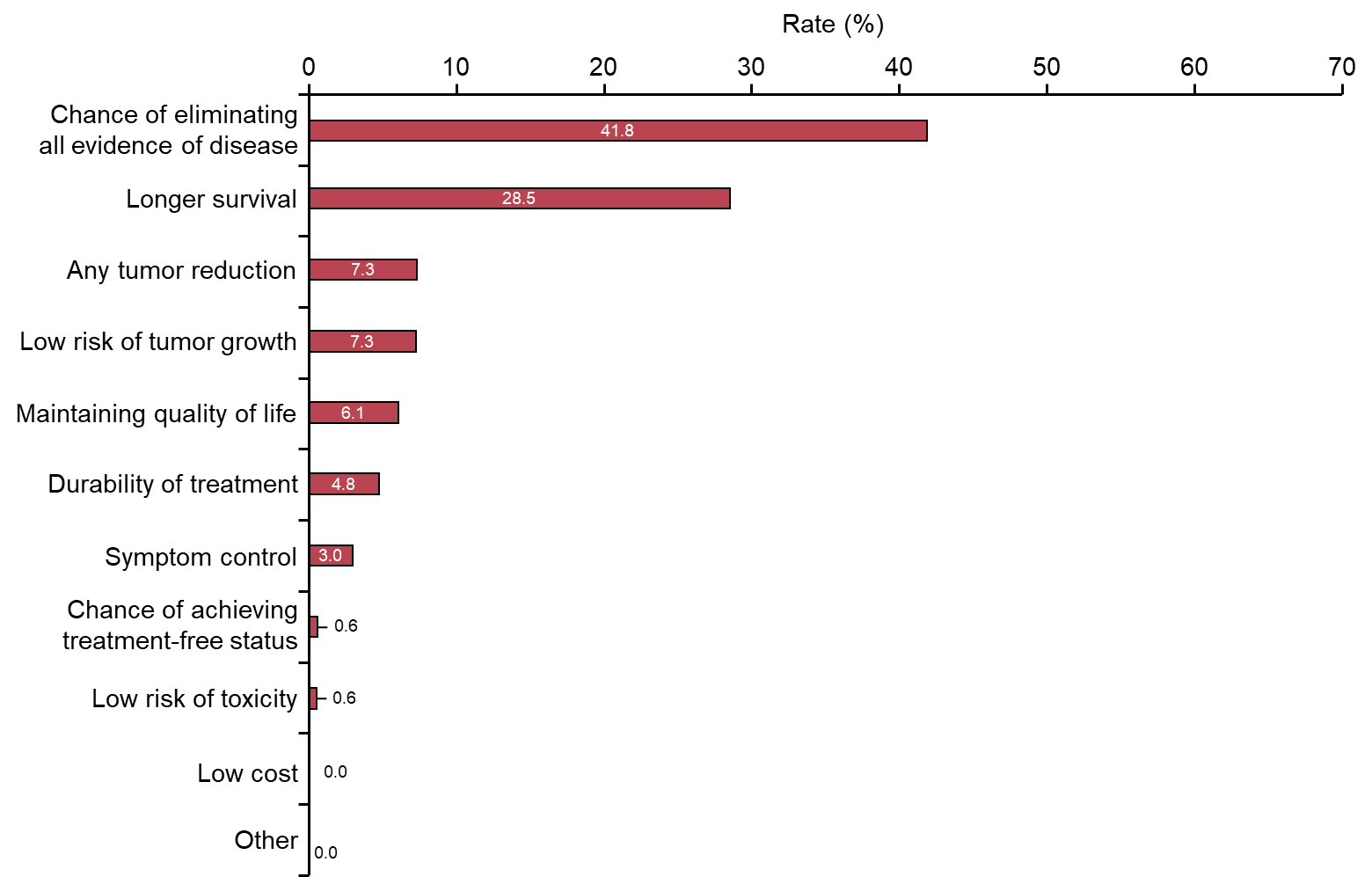
B.**

Supplementary figure S3: Concerns about systemic therapy for patients (A) and physicians (B) (only first-ranked responses)

**
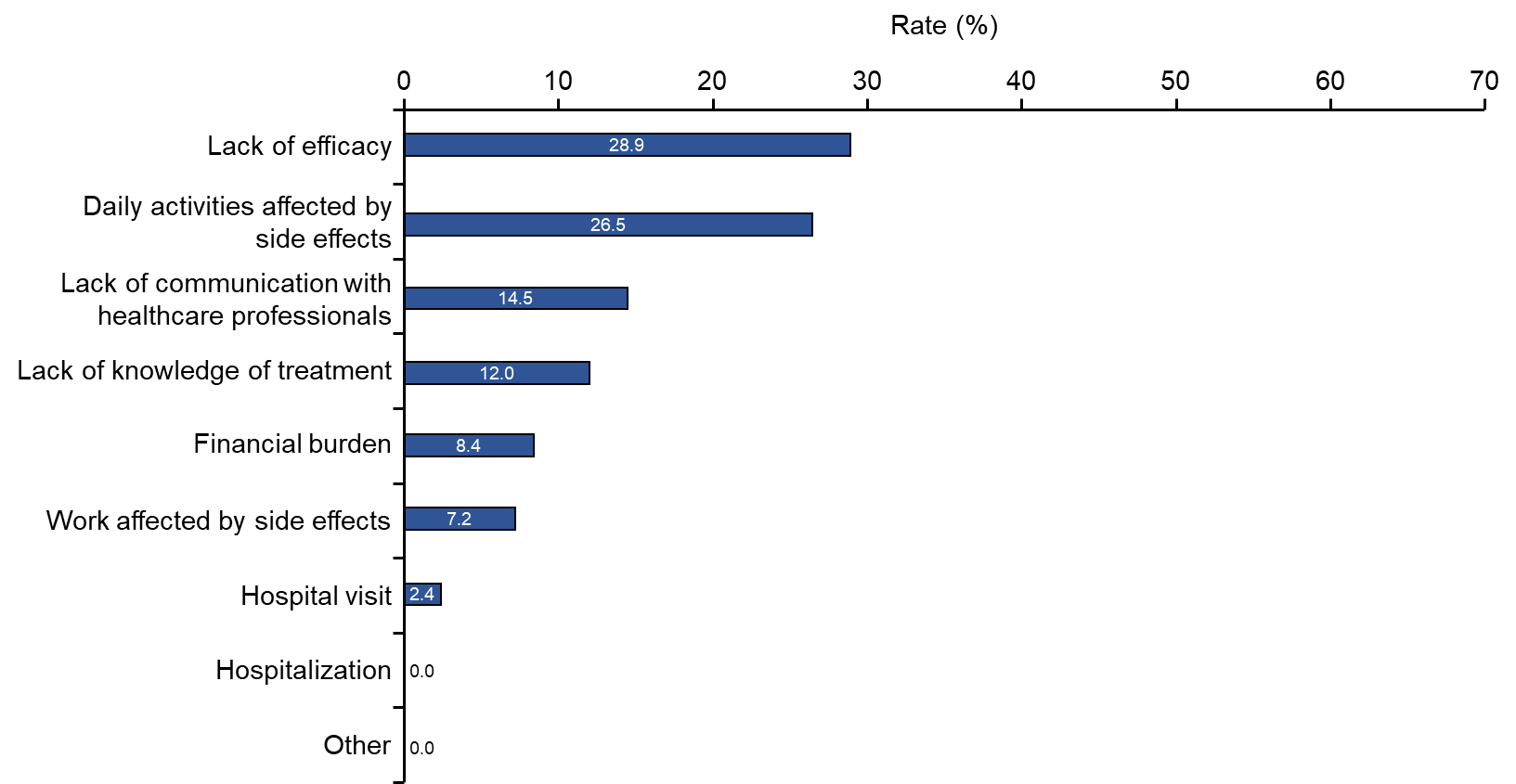
A.**

**
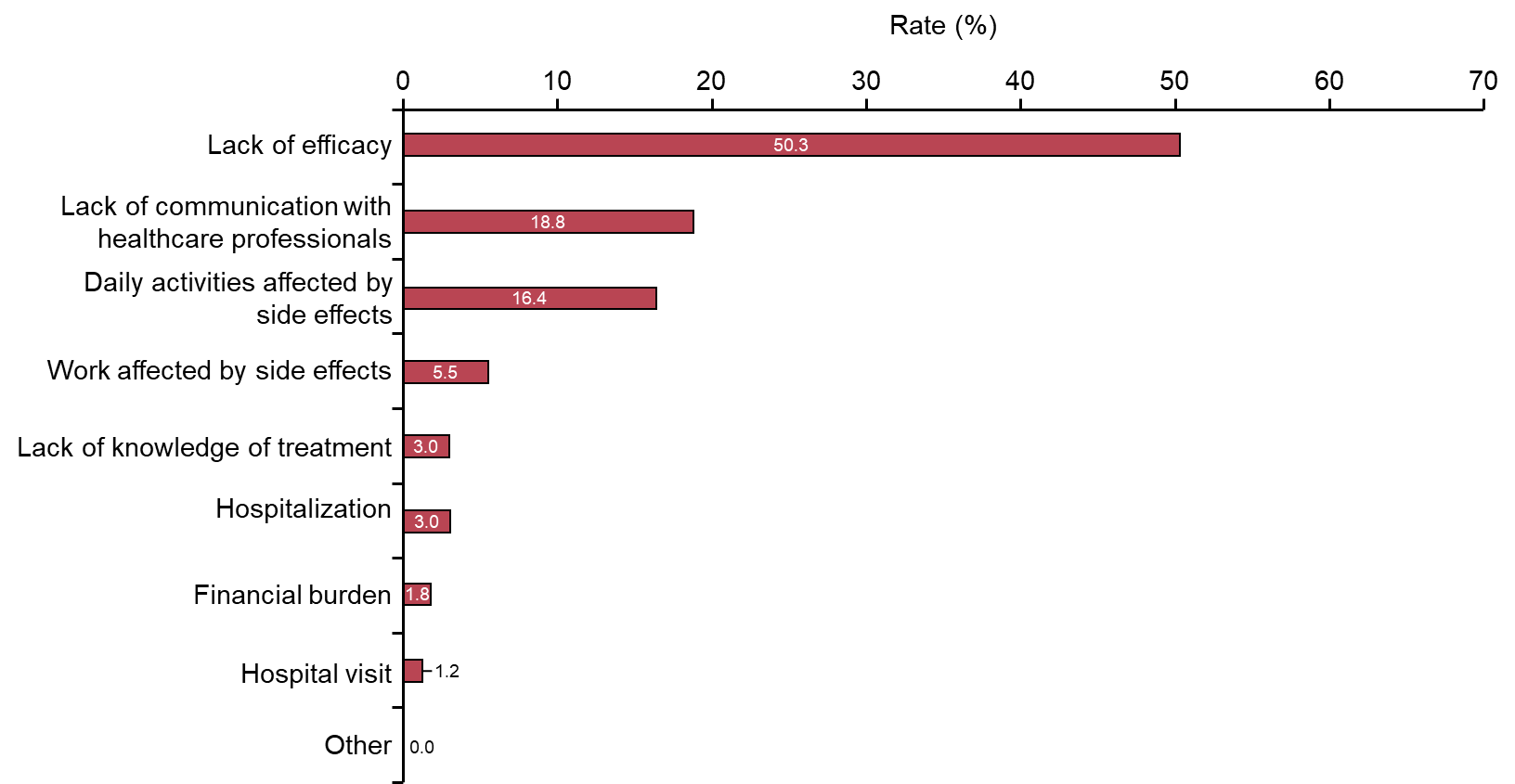
B.**

**Supplementary Figure S4: Distressing adverse events for patients (only first-ranked responses)**

**
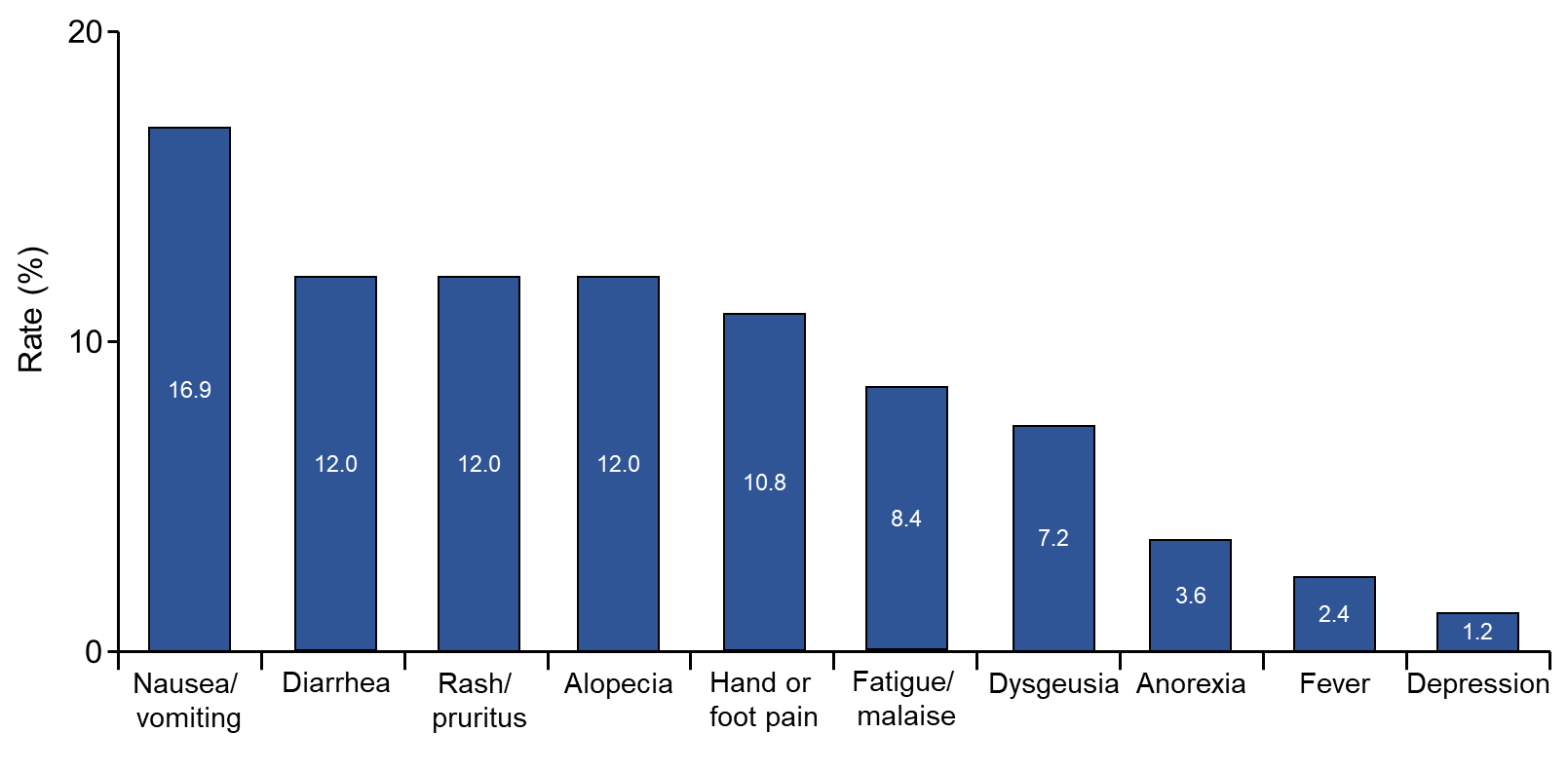
**
